# Relationship of common hemodynamic and respiratory target parameters with brain tissue oxygen tension in the absence of hypoxemia or hypotension after cardiac arrest: A post-hoc analysis of an experimental study using a pig model

**DOI:** 10.1371/journal.pone.0245931

**Published:** 2021-02-04

**Authors:** Yong Hun Jung, Kamoljon Shamsiev, Najmiddin Mamadjonov, Kyung Woon Jeung, Hyoung Youn Lee, Byung Kook Lee, Byung Soo Kang, Tag Heo, Yong Il Min

**Affiliations:** 1 Department of Emergency Medicine, Chonnam National University Hospital, Gwangju, Republic of Korea; 2 Department of Emergency Medicine, Chonnam National University Medical School, Gwangju, Republic of Korea; 3 Department of Medical Science, Chonnam National University Graduate School, Gwangju, Republic of Korea; 4 Department of Medical Science, College of Medicine, Chosun University, Gwangju, Republic of Korea; Fondazione IRCCS Policlinico San Matteo, ITALY

## Abstract

Brain tissue oxygen tension (PbtO_2_)-guided care, a therapeutic strategy to treat or prevent cerebral hypoxia through modifying determinants of cerebral oxygen delivery, including arterial oxygen tension (PaO_2_), end-tidal carbon dioxide (ETCO_2_), and mean arterial pressure (MAP), has recently been introduced. Studies have reported that cerebral hypoxia occurs after cardiac arrest in the absence of hypoxemia or hypotension. To obtain preliminary information on the degree to which PbtO_2_ is responsive to changes in the common target variables for PbtO_2_-guided care in conditions without hypoxemia or hypotension, we investigated the relationships between the common target variables for PbtO_2_-guided care and PbtO_2_ using data from an experimental study in which the animals did not experience hypoxemia or hypotension after resuscitation. We retrospectively analyzed 170 sets of MAP, ETCO_2_, PaO_2_, PbtO_2_, and cerebral microcirculation parameters obtained during the 60-min post-resuscitation period in 10 pigs resuscitated from ventricular fibrillation cardiac arrest. PbtO_2_ and cerebral microcirculation parameters were measured on parietal cortices exposed through burr holes. Multiple linear mixed effect models were used to test the independent effects of each variable on PbtO_2_. Despite the absence of arterial hypoxemia or hypotension, seven (70%) animals experienced cerebral hypoxia (defined as PbtO_2_ <20 mmHg). Linear mixed effect models revealed that neither MAP nor ETCO_2_ were related to PbtO_2_. PaO_2_ had a significant linear relationship with PbtO_2_ after adjusting for significant covariates (P = 0.030), but it could explain only 17.5% of the total PbtO_2_ variance (semi-partial R^2^ = 0.175; 95% confidence interval, 0.086–0.282). In conclusion, MAP and ETCO_2_ were not significantly related to PbtO_2_ in animals without hypoxemia or hypotension during the early post-resuscitation period. PaO_2_ had a significant linear association with PbtO_2_, but its ability to explain PbtO_2_ variance was small.

## Introduction

Despite advances in the treatment of cardiac arrest, most cardiac arrest survivors fail to achieve meaningful neurological recovery due to brain injury [1–3]. Brain injury after cardiac arrest results not only from interruption of cerebral blood flow (CBF) during circulatory arrest but also from various pathophysiological derangements after restoration of spontaneous circulation (ROSC). Several studies have suggested associations between perturbations in cerebral oxygen delivery after ROSC and worse neurological outcome after cardiac arrest [4–10]. In line with these studies [4–10], recent post-cardiac arrest care guidelines have emphasized the importance of maintaining adequate arterial oxygen tension (PaO_2_), carbon dioxide tension (PaCO_2_), and mean arterial pressure (MAP) to ensure sufficient cerebral oxygenation, which in turn prevents secondary hypoxic-ischemic brain injury [11, 12].

Tissue oxygen tension, the partial pressure of oxygen within the interstitial space of a tissue, has been measured in various clinical and laboratory settings to assess the adequacy of tissue oxygenation. Currently, two types of oxygen sensors are widely used to measure tissue oxygen tension: polarographic electrodes and optical sensors. Polarographic electrodes measure oxygen tension based on the electrical current produced when an anode and cathode submerged in an electrolyte solution come into contact with oxygen [13]. Optical sensors measure the oxygen tension resulting from luminescence quenching in the presence of oxygen [14]. Brain tissue oxygen tension (PbtO_2_) monitoring is performed by placing an oxygen sensor within brain tissue through a burr hole. This technique is an established method for direct real-time monitoring of cerebral oxygenation. It has been used as a neuromonitoring modality in the management of patients with severe traumatic brain injury. In patients with severe traumatic brain injury, low PbtO_2_ levels have been shown to increase the risk of hypoxic-ischemic brain injury and the likelihood of poor outcomes [15–18]. Recently, PbtO_2_-guided care, a therapeutic strategy to treat or prevent cerebral hypoxia through modifying determinants of cerebral oxygen delivery, including PaO_2_, end-tidal carbon dioxide (ETCO_2_), and MAP, has been introduced in post-cardiac arrest care [19–21]. Although it appears to be a promising strategy, further information regarding PbtO_2_ levels after cardiac arrest is needed before PbtO_2_-guided care can be trialed or widely implemented in clinical post-cardiac arrest care. In several studies using PbtO_2_ monitoring, despite maintaining adequate arterial oxygenation and perfusion pressure, episodes of cerebral hypoxia frequently occurred after resuscitation from cardiac arrest [20–22]. It is evident that correcting hypoxemia and/or hypotension can increase PbtO_2_ levels [23–26]. However, no studies have evaluated the impact of increasing PaO_2_ and/or MAP on PbtO_2_ levels in non-hypoxemic, non-hypotensive cardiac arrest patients during the early post-resuscitation period.

In this study, we sought to determine the extent to which the common target variables for PbtO_2_-guided care, including PaO_2_, ETCO_2_, and MAP, are related to PbtO_2_ in the early post-resuscitation period using data from an experimental study conducted using a pig model of cardiac arrest in which the animals did not experience hypoxemia or hypotension during the early post-resuscitation period. The results of this analysis would provide preliminary information on the degree to which PbtO_2_ is responsive to changes in the common target variables for PbtO_2_-guided care and, thus, would help identify an optimal management protocol for PbtO_2_-guided care. We hypothesized that in conditions without hypoxemia or hypotension after cardiac arrest, PaO_2_, ETCO_2_, and MAP would be closely related to the PbtO_2_.

## Materials and methods

This study was a post-hoc analysis of data derived from a previous study investigating the effects of pralidoxime administration during cardiopulmonary resuscitation (CPR) on hemodynamics and cerebral microcirculation after ROSC in 20 healthy Yorkshire/Landrace cross pigs weighing 24.7 ± 1.7 kg [27]. In this study, data from 10 animals that received only standard advanced cardiovascular life support (control group) were included. All experiments were approved by the Animal Care and Use Committee of Chonnam National University Hospital (CNUH IACUC-18004) and performed in accordance with the National Institutes of Health Guide for the Care and Use of Laboratory Animals. The investigators who performed the experiments had completed an Institutional Animal Care and Use Committee training course on animal care and handling. All surgical interventions were performed under sevoflurane anesthesia, and every effort was made to prevent unnecessary suffering of the animals.

### Animal preparation

The animals were fasted overnight, but they were given water ad libitum. After an intramuscular injection of ketamine (20 mg/kg) and xylazine (2.2 mg/kg), they were placed in a supine position, and their trachea was intubated with a 7.0-mm internal diameter tracheal tube. The animals were anesthetized using an anesthesia machine with a 70/30 mixture of nitrous oxide/oxygen and sevoflurane. During surgical intervention, sevoflurane was titrated to maintain adequate anesthetic depth, measured by the absence of reflex withdrawal and no change in heart rate, arterial pressure, or respiratory rate. They were mechanically ventilated in a volume control mode with the following settings: tidal volume, 10 ml/kg; respiratory rate, 14 breaths/min; positive end-expiratory pressure, 5 cmH_2_O; inspiratory/expiratory ratio, 1:2; and fraction of inspired oxygen (FiO_2_), 0.3. ETCO_2_ was monitored using a sidestream capnography monitor (B40 Patient Monitor; GE Healthcare, Chalfont St. Giles, UK), and the respiratory rate was subsequently adjusted to maintain normocapnia. A 6.0-F introducer sheath was advanced through the right external jugular vein for pacemaker wire insertion and right atrial pressure monitoring. A 7.0-F saline-filled catheter was advanced through the left femoral artery for arterial pressure monitoring and blood sampling. Another 7.0-F saline-filled catheter was inserted into the left internal jugular vein and advanced toward the skull base for jugular venous blood sampling. After subcutaneous infiltration of 2% lidocaine solution, a scalp incision was made to expose the right and left parietal bones. A 10-mm diameter burr hole was drilled bilaterally through the parietal bone (10-mm lateral and 10-mm posterior to the intersection of the coronal and sagittal sutures). The dura underneath the burr hole was opened to visualize the pial vessels. Three adhesive electrodes were applied on limbs to record the electrocardiogram. Normal saline was infused to maintain a mean right atrial pressure of 5–10 mmHg. Throughout the experiment, the rectal temperature was maintained at 38°C using a heating blanket.

### Experimental protocol

**Fig 1:**
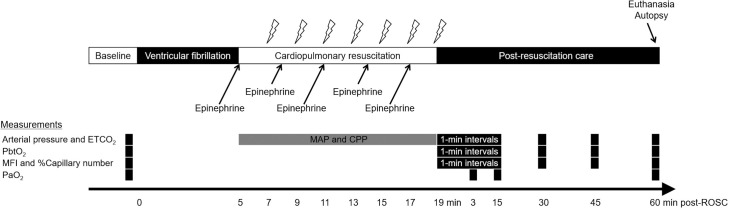
Experimental timeline. After 5 min of untreated ventricular fibrillation, cardiopulmonary resuscitation was initiated using a mechanical chest compression device. The lightning marks indicate the onset of a 10-s pause in chest compression for rhythm analysis and a 150-J electric shock, if indicated. After ROSC, the animals were observed for 60 min in a simulated intensive care setting. Arterial pressure, ETCO_2_, MFI, %Capillary number, and PbtO_2_ were sampled at pre-arrest baseline, 1-min intervals for 15 min after ROSC, and 30, 45, and 60 min after ROSC. Arterial blood gases were measured at pre-arrest baseline and 3, 15, and 60 min after ROSC. MAP and CPP during cardiopulmonary resuscitation were sampled 2 min after the initiation of cardiopulmonary resuscitation since most of the animals (80%) achieved ROSC 2–4 min after cardiopulmonary resuscitation. ETCO_2_, end-tidal carbon dioxide; PbtO_2_, brain tissue oxygen tension, MFI, microvascular flow index; %Capillary number, percent of counted number of perfused capillaries relative to that at pre-arrest baseline; PaO_2_, partial pressure of oxygen in arterial blood; MAP, mean arterial pressure; CPP, coronary perfusion pressure; ROSC, restoration of spontaneous circulation.

After baseline measurements were obtained, an electrical current (60 Hz and 30 mA alternating current) was applied through the pacemaker wire placed in the right ventricle to induce ventricular fibrillation (VF). Mechanical ventilation was discontinued immediately after the onset of VF. After 5 min of untreated VF (Fig 1), external mechanical chest compressions (Life-Stat; Michigan Instruments, Grand Rapids, MI, USA) with a compression depth comparable to 20% of the anteroposterior chest diameter and a rate of 100 compressions/min were initiated. Simultaneously, ventilation was provided with high-flow oxygen (14 l/min) at a rate of 10 breaths/min using a volume-marked bag devised by Cho et al [28]. During CPR, epinephrine (0.02 mg/kg) was administered intravenously every 3 min, and defibrillation was attempted with a single biphasic 150-J electric shock delivered between the right infraclavicular area and the cardiac apex every 2 min, if indicated. These procedures were continued until ROSC was achieved or until 14 min had elapsed since the initiation of CPR after which CPR was discontinued.

After ROSC, the animals were observed for 60 min in a simulated intensive care setting. General anesthesia with sevoflurane was provided throughout the 60-min period. Immediately after ROSC, mechanical ventilation was resumed with an FiO_2_ of 1.0; the other ventilator settings were unchanged. Fifteen minutes after achieving ROSC, the ventilatory rate was adjusted to maintain an ETCO_2_ of 45 mmHg. Normal saline (10 ml/kg/h) was intravenously infused to maintain normovolemia during the 60-min period, but no hemodynamic drugs were given during this period. We predetermined the humane endpoints for euthanasia (systolic arterial pressure <60 mmHg, heart rate <40 beats/min, or seizure), but none of the animals reached the endpoints until completion of the experimental protocol. Immediately after completing the experimental protocol, the animals were euthanized with a rapid bolus of 40 mEq potassium chloride under general anesthesia.

### Measurements

Arterial pressure and right atrial pressure were continuously monitored (CS/3 CCM; Datex-Ohmeda, Helsinki, Finland) and stored on a personal computer using a data collection software (S/5 Collect software, Datex-Ohmeda, Helsinki, Finland). Coronary perfusion pressure during CPR was calculated from the differences in time-coincident aortic end-diastolic and right atrial pressures. PbtO_2_ and cerebral microcirculatory blood flow were assessed in the right and left parietal cortices. The measurement site (right or left) was randomized and counterbalanced. PbtO_2_, the study primary outcome, was measured using an optical oxygen sensor (DP-PSt7; PreSens-Precision Sensing GmbH, Regensburg, Germany), which was placed directly on the surface of the parietal cortex. To assess the responsiveness of PbtO_2_ to changes in FiO_2_ at pre-arrest baseline, FiO_2_ was increased from 0.3 to 1.0, maintained at 1.0 until the PbtO_2_ level reached a plateau, and then decreased to 0.3. Cerebral hypoxia was defined as PbtO_2_ <20 mmHg [29], and the duration of exposure to cerebral hypoxia was determined for each animal. Cerebral cortical microcirculation images were recorded using a hand-held digital microscope (G-Scope G5; Genie Tech, Seoul, Korea) placed over the burr hole. The microscope provided ×250 magnification and showed an area of interest of approximately 1800 × 1000 μm^2^. Two investigators analyzed the cerebral microcirculation videos and quantitated the microvascular flow index (MFI) of vessels <20 μm in diameter (representing primarily capillaries) by consensus [30, 31]. They also counted the number of perfused capillaries as previously described by Serné et al [32]. The number of perfused capillaries after ROSC was expressed as a percent of the counted number of perfused capillaries relative to that at the pre-arrest baseline (%Capillary number) to correct for its substantial inter-animal variation. Arterial pressure, ETCO_2_, MFI, %Capillary number, and PbtO_2_ were sampled at pre-arrest baseline, 1-min intervals for 15 min after ROSC, and 30, 45, and 60 min after ROSC. Arterial blood gases were measured at pre-arrest baseline and 3, 15, and 60 min after ROSC.

### Statistical analysis

Data were analyzed using R version 3.3.3 (R Foundation for Statistical Computing, Vienna, Austria) and T&F program version 3.0 (YooJin BioSoft, Goyang, Republic of Korea). Continuous variables, presented as mean ± standard deviation or median values with interquartile ranges, were examined for normality using the Shapiro–Wilk test. We used multiple linear mixed effect models to test the independent effects of common target variables for PbtO_2_-guided care, including PaO_2_, ETCO_2_, and MAP, on the PbtO_2_ during the 60-min post-resuscitation period. In addition, we also tested the independent effects of cerebral microcirculation variables, including MFI and %Capillary number, on the PbtO_2_. Since arterial blood gases were measured only at three timepoints (3, 15, and 60 min) after ROSC, PaO_2_ data were not available for several timepoints at which the other target variables were sampled. In the original study [27], we found that PaO_2_ levels linearly increased over time during the 60-min post-resuscitation period. Thus, PaO_2_ values at unmeasured timepoints were derived using linear interpolation and extrapolation methods. Mixed models included each predictor variable (PaO_2_, ETCO_2_, MAP, MFI, and %Capillary number) and time as fixed effect covariates with random effects of intercept and slope for time and each predictor variable. The hemodynamic, blood gas, and cerebral measurement data obtained at pre-arrest baseline and CPR data, including the number of electric shocks, number of epinephrine administrations, duration of CPR, and coronary perfusion pressure and MAP during CPR, were independently examined to evaluate their effectiveness in determining the PbtO_2_. Significant variables (P value cutoff = 0.05) were further used for covariate adjustment in the mixed effect models. Semi-partial R-squared values of the fixed effects were computed for predictor variables and time using the r2glmm package of R software. A two-tailed P-value of <0.05 was considered statistically significant.

## Results

**Fig 2:**
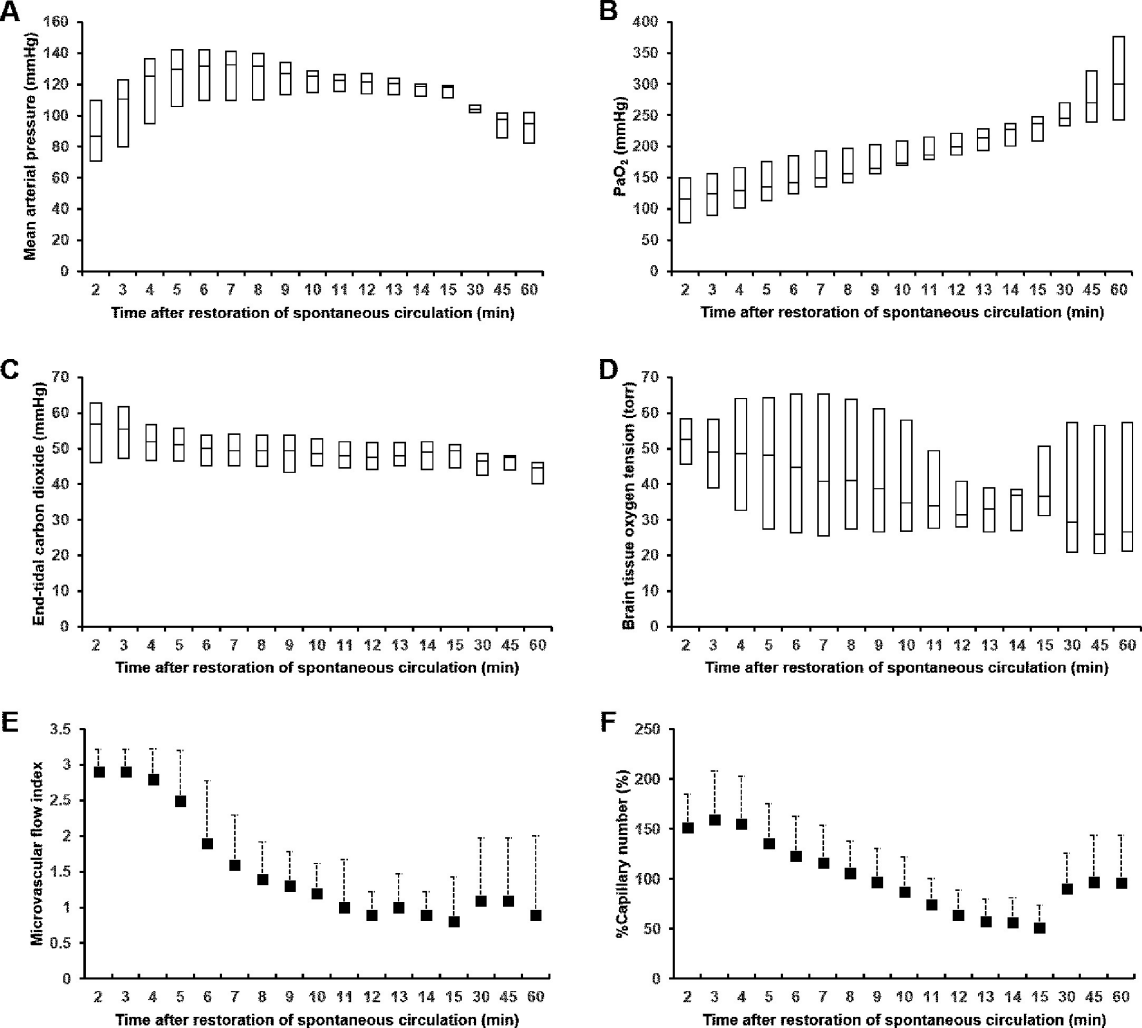
Mean arterial pressure (A), arterial oxygen tension (B), end-tidal carbon dioxide (C), brain tissue oxygen tension (D), microvascular flow index (E), and %Capillary number (E) after the restoration of spontaneous circulation. Data are presented as medians with interquartile ranges (A–D) or mean ± standard deviation (E and F). PaO_2_, partial pressure of oxygen in arterial blood; %Capillary number, percent of counted number of perfused capillaries relative to that at the pre-arrest baseline.

Pre-arrest baseline measurements were within normal limits, as shown in Table 1. The PbtO_2_ was 31.4 ± 6.6 torr at an FiO_2_ of 0.3. Increasing the FiO_2_ to 1.0 resulted in a marked increase in the PbtO_2_ to 63.8 ± 18.1 torr. All animals achieved ROSC after 4 (2–5) min of CPR and survived the 60-min observation period. Thus, a total of 170 sets of MAP, ETCO_2_, PaO_2_, MFI, %Capillary number, and PbtO_2_ data were collected during the 60-min post-resuscitation period (17 times in 10 animals) and included in the analyses. Fig 2 shows MAP, ETCO_2_, PaO_2_, MFI, %Capillary number, and PbtO_2_ data during the 60-min post-resuscitation period. Immediately after ROSC, MAP increased rapidly, reaching a plateau within 5 min. It decreased progressively after 15 min, but it was maintained >65 mmHg. ETCO_2_ and PbtO_2_ progressively decreased during the 60-min period, while PaO_2_ progressively increased. Immediately after ROSC, cerebral cortices became hyperemic; hence, there were more perfused capillaries than those at baseline for all animals. After a brief hyperemic period, cerebral cortices turned pale and the %Capillary number and MFI decreased progressively over the next 10 min. S1 Movie shows a representative video of the cerebral microcirculation. Despite the absence of arterial hypoxemia, hypocarbia, or hypotension, seven (70%) animals had cerebral hypoxia for 12.1 (6.6–33.5) min. Table 2 shows the fixed effects of time and each predictor variable on PbtO_2_ in linear mixed effect models. In the linear mixed effect models, PaO_2_ levels had a significant linear relationship with PbtO_2_ after adjusting for significant covariates (semi-partial R^2^ = 0.175; 95% confidence interval [CI], 0.086–0.282, P = 0.030), but neither MAP nor ETCO_2_ were related to PbtO_2_. Although the cerebral microcirculation variables were significantly associated with PbtO_2_, their ability to explain PbtO_2_ variance was limited. When adjusted for significant covariates, the MFI and %Capillary number explained 14.3% (semi-partial R^2^ = 0.143; 95% CI = 0.062–0.247, P = 0.023) and 11.3% (semi-partial R^2^ = 0.113; 95% CI = 0.040–0.212, P = 0.048) of the total PbtO_2_ variance, respectively.

**Table 1:** Pre-arrest baseline measurement and cardiopulmonary resuscitation data.

Weight (kg)	25.3 ± 1.3
Systolic arterial pressure (mmHg)	134 ± 8
Diastolic arterial pressure (mmHg)	90 ± 11
Mean arterial pressure (mmHg)	108 ± 9
Systolic right atrial pressure (mmHg)	13 (10–13)
Diastolic right atrial pressure (mmHg)	5 (5–6)
Mean right atrial pressure (mmHg)	9 ± 1
Heart rate (/min)	101 (88–108)
End-tidal carbon dioxide (mmHg)	45 (42–45)
Rectal temperature (°C)	38.0 ± 0.4
pH	7.502 ± 0.031
PaCO_2_ (mmHg)	44.1 ± 3.4
PaO_2_ (mmHg)	126.4 ± 21.4
HCO_3_^-^ (mmol/l)	34.5 ± 2.3
Lactate (mmol/l)	0.87 ± 0.37
SaO_2_ (%)	99.0 (99.0–99.3)
PbtO_2_ at FiO_2_ 0.3 (torr)	31.4 ± 6.6
PbtO_2_ at FiO_2_ 1.0 (torr)	63.8 ± 18.1
Microvascular flow index*	3
Number of perfused capillaries (n)	13 ± 3
Number of electric shocks (n)	1 (1–2)
Number of epinephrine administrations (n)	2 (1–2)
Duration of CPR (min)	4 (2–5)
Mean arterial pressure after 2 min of CPR (mmHg)	49.9 ± 10.2
Coronary perfusion pressure after 2 min of CPR (mmHg)	17.5 ± 4.8

Data are presented as medians with interquartile ranges or mean ± standard deviation.

* The microvascular flow index at pre-arrest baseline was 3 in all animals. PaCO_2_, partial pressure of carbon dioxide in arterial blood; PaO_2_, partial pressure of oxygen in arterial blood; HCO_3_^-^, bicarbonate; SaO_2_, oxygen saturation in arterial blood; PbtO_2_, brain tissue oxygen tension; FiO_2_, fraction of inspired oxygen; CPR, cardiopulmonary resuscitation.

**Table 2:** Fixed effects of time and each predictor variable on brain tissue oxygen tension in mixed effect models.

Variable	Coefficient	SE	P-value	Semi-partial R^2^	95% CI
Time	-0.165	0.181	0.384	0.038	0.002–0.112
Mean arterial pressure (mmHg)	-0.090	0.155	0.578	0.028	0.001–0.095
Time*	-0.177	0.180	0.349	0.044	0.004–0.121
Mean arterial pressure (mmHg)*	-0.102	0.152	0.522	0.031	0.001–0.101
Time	0.153	0.320	0.643	0.022	0–0.086
PaO_2_	-0.120	0.056	0.053	0.271	0.170–0.380
Time*	0.192	0.324	0.567	0.026	0.001–0.093
PaO_2_*	-0.135	0.055	0.030	0.175	0.086–0.282
Time	-0.177	0.226	0.452	0.033	0.001–0.103
End-tidal carbon dioxide (mmHg)	0.058	0.298	0.846	0.001	0–0.033
Time*	-0.181	0.227	0.442	0.034	0.002–0.106
End-tidal carbon dioxide (mmHg)*	0.037	0.299	0.901	0	0–0.031
Time	-0.135	0.223	0.559	0.023	0–0.087
Microvascular flow index	5.729	2.161	0.025	0.142	0.060–0.245
Time*	-0.132	0.221	0.564	0.022	0–0.085
Microvascular flow index*	5.777	2.125	0.023	0.143	0.062–0.247
Time	-0.119	0.238	0.628	0.018	0–0.077
%Capillary number	10.623	4.688	0.049	0.127	0.050–0.228
Time*	-0.122	0.235	0.616	0.019	0–0.079
%Capillary number*	10.507	4.632	0.048	0.113	0.040–0.212

* Baseline end-tidal carbon dioxide and rectal temperature were used to adjust the mixed effect model. SE, standard error; CI, confidence interval; PaO_2_, partial pressure of oxygen in arterial blood; %Capillary number, percent of counted number of perfused capillaries relative to that at the pre-arrest baseline.

## Discussion

Using data from an experimental study in which the animals did not experience hypoxemia or hypotension during the early post-resuscitation period, this study showed that PaO_2_, but not MAP nor ETCO_2_, had a significant relationship with PbtO_2_. To the best of our knowledge, this is the first study to examine the relationship between key determinants of cerebral oxygen delivery and PbtO_2_ in non-hypoxemic, non-hypotensive conditions after cardiac arrest.

Several studies reported the frequent occurrence of cerebral hypoxia after ROSC despite adequate arterial oxygenation and perfusion pressure [20–22]. In a study that included 14 comatose cardiac arrest patients who underwent invasive neuromonitoring [22], 7 patients (50%) had a mean PbtO_2_ level ≤ 20 torr, which is a widely accepted hypoxic threshold [29]. Consistent with these studies [20–22], 70% of the animals in this study experienced PbtO_2_ values < 20 torr in the absence of hypoxemia or hypotension. This finding indicates that a significant percentage of cardiac arrest patients may experience secondary hypoxic insults to the brain if the initial treatments are solely based on PaO_2_ and MAP. PbtO_2_ monitoring, although highly invasive, has been repeatedly reported as a safe procedure [16, 20, 22, 24, 33, 34]. This technique could help identify patients with ongoing cerebral hypoxia after ROSC who would not otherwise be detected.

Cerebral oxygenation can be monitored noninvasively by near-infrared spectroscopy (NIRS), which estimates regional tissue oxygenation by using light waves of near-infrared wavelength to penetrate the scalp, skull, and brain. Although NIRS is a non-invasive and easy-to-use technique for monitoring cerebral oxygenation, it measures the oxygen saturation of combined arterial, venous, and microcirculatory blood compartments in both cerebral and extracerebral tissue [35–37], which limits its accuracy and reliability for reflecting PbtO_2_ levels [38, 39]. However, technological advances continue to improve the accuracy and usefulness of NIRS as a non-invasive tool for estimating the PbtO_2_ [40]. Consequently, NIRS technology has the future potential to serve as a reliable and non-invasive surrogate of PbtO_2_.

Although data regarding the significance of reduced PbtO_2_ levels on clinical outcomes in cardiac arrest patients are unavailable, several studies involving patients with severe traumatic brain injury have suggested associations between reduced PbtO_2_ levels and poor outcomes [15–18]. In a study investigating the relationship between low PbtO_2_ values and outcomes in 101 comatose head-injured patients [15], the depth and duration of low PbtO_2_ values during the first 24 h after injury correlated with unfavorable neurological outcomes and death at 6 mon. Studies have also suggested that reduced PbtO_2_ can be a modifiable therapeutic target for neurological recovery [21, 33]. In a pig model of opioid overdose cardiac arrest, Elmer et al. reported that PbtO_2_-guided care, compared with standard care, reduced the duration of exposure to PbtO_2_ levels below the hypoxic threshold [21]. A phase 2 randomized trial including 119 patients with severe traumatic brain injury conducted by Okonkwo et al. compared a treatment protocol based on intracranial pressure (ICP) plus PbtO_2_ monitoring, to a protocol based on ICP monitoring alone [33]. The results demonstrated that the treatment protocol based on PbtO_2_ plus ICP monitoring reduced the time of exposure to cerebral hypoxia, with a trend toward lower mortality and favorable neurological recovery.

MAP is the main determinant of cerebral perfusion pressure, which is the driving force of CBF. The lack of a significant relationship between MAP and PbtO_2_ in this study is best explained by the presence of cerebral autoregulation. In our data, the overall MAP values were above the suggested autoregulation threshold of 60–75 mmHg [24, 41, 42]. Several studies have suggested that the cerebral autoregulation mechanism, although it is often right shifted, operates in most cardiac arrest patients [20, 43]. In a study that included 51 comatose cardiac arrest patients who underwent MAP and regional cerebral oxygen saturation monitoring during the first 24 h of intensive care unit stay [43], cerebral autoregulation was preserved in about two thirds of the patients, whereas it was right-shifted in one third of the patients. In a study that assessed the relationship between MAP and brain tissue oxygenation in 10 cardiac arrest survivors [20], MAP was positively associated with PbtO_2_ when MAP values were below the optimal MAP, but not when MAP values were above the optimal MAP, determined using the pressure reactivity index (89 ± 11 mmHg). Our finding suggests that in non-hypotensive cardiac arrest patients, MAP augmentation may not be able to influence PbtO_2_ considerably. This finding is consistent with that of a recent randomized clinical trial that compared targeted low-normal (65–75 mmHg) and high-normal MAP (80–100 mmHg) in 123 comatose cardiac arrest patients [44], in which despite a clear separation in MAP between the two MAP target groups, the regional cerebral oxygen saturation (measured by using NIRS monitoring), neurobiomarkers (neuron-specific enolase and S100B), and clinical outcomes did not differ between the two groups.

Under physiological conditions, changes in PaCO_2_ influence CBF considerably [45]. ETCO_2_, a non-invasive surrogate of PaCO_2_, is widely used to guide ventilator management in critically ill patients and is also related to CBF [46]. A study investigating ETCO_2_ response curves for middle cerebral artery blood flow velocity (measured by using the transcranial Doppler technique) in 31 normal subjects showed that ETCO_2_ was well correlated with the middle cerebral artery blood flow velocity [46]. Previous studies reported that cerebrovascular reactivity to PaCO_2_ was preserved in patients resuscitated from cardiac arrest [47, 48]. In a study that investigated cerebrovascular reactivity to changes in PaCO_2_ for 24 h after intensive care unit admission in 10 comatose out-of-hospital cardiac arrest patients [48], the mean percentage change in middle cerebral artery blood flow velocity per mmHg PaCO_2_ change (3.6 ± 2.9%) was comparable to that reported in healthy subjects [49]. However, in contrast to these studies [47, 48], our study did not show a significant relationship between ETCO_2_ and PbtO_2_. The reason for this lack of a significant relationship between ETCO_2_ and PbtO_2_ is unclear, but it may be due to the blunted cerebrovascular reactivity occurring in the early post-ischemic period. Several studies have suggested that cerebrovascular reactivity to PaCO_2_ is markedly blunted in the early post-ischemic period [50, 51]. In a dog model of post-ischemic cerebral hypoperfusion, cerebrovascular reactivity was markedly blunted after recovery from 15 min of global ischemia, while cerebral autoregulation was maintained [50]. In a study that evaluated the reactivity of PbtO_2_ to changes in PaCO_2_ over time in patients with severe head injury [52], the reactivity of PbtO_2_ was initially abolished, but it recovered within the first two days after sustaining an injury.

Among the common target variables for PbtO_2_-guided care that we investigated, only PaO_2_ had a significant relationship with PbtO_2_ in this study. This finding is consistent with that reported in other studies [19, 34]. In rats subjected to asphyxial cardiac arrest, reduced cortical PbtO_2_ increased with an increase in FiO_2_; however, it did not respond to increasing MAP with epinephrine infusion [19]. Our findings, together with the findings reported by the above-mentioned studies [19, 34], suggest that to increase PbtO_2_ in non-hypoxemic, non-hypotensive cardiac arrest patients, care should primarily focus on increasing PaO_2_ rather than increasing ETCO_2_ or MAP. However, the following points make the therapeutic efficacy of increasing PaO_2_ questionable. Although the PaO_2_ level had a significant linear association with the PbtO_2_ level, it explained only 17.5% of the total PbtO_2_ variance. This finding suggests that the extent to which one can increase PbtO_2_ by increasing PaO_2_ may not be high enough to be clinically useful. Its limited ability to explain the PbtO_2_ variance may be related to impaired cerebral microcirculation, as evidenced by the reduced MFI and number of perfused capillaries during the post-resuscitation period. In several studies, the response of PbtO_2_ to supplemental oxygen diminished in the presence of reduced CBF [53, 54]. A study investigating the role of regional CBF in determining the response of PbtO_2_ to induced hyperoxia in 83 patients with traumatic brain injury showed that the increase in PbtO_2_ induced by hyperoxia was smaller when regional CBF was < 20 ml/100 g/min than when it was > 20 ml/100g/min [53]. In the presence of reduced PbtO_2_ response to supplemental oxygen, it would be difficult to treat cerebral hypoxia by increasing FiO_2_ without exposing the patients to arterial hyperoxemia, which has repeatedly been linked with poor outcomes after cardiac arrest [8, 55, 56].

In this study, the perfusion of cerebral capillaries, which serve as the ultimate exchange vessels for oxygen and vital substrates, explained only a small proportion of PbtO_2_ variance. The limited capability of the MFI and %Capillary number to explain PbtO_2_ variance, in addition to that of PaO_2_, suggests that factors other than cerebral perfusion and blood oxygenation affect the PbtO_2_ level. Recent studies have suggested that the diffusion limitation of oxygen delivery resulting from extensive perivascular edema and endothelial swelling is a plausible mechanism for cerebral hypoxia [22, 57]. A study assessing the relationship of the jugular venous oxygen tension-PbtO_2_ gradient with cerebral perfusion pressure in 14 comatose cardiac arrest patients showed that increased cerebral perfusion pressure was associated with a decrease in the jugular venous oxygen tension-PbtO_2_ gradient in patients without cerebral hypoxia (PbtO_2_ > 20 mmHg) [22]. However, patients with cerebral hypoxia (PbtO_2_ ≤ 20 mmHg) exhibited an increased jugular venous oxygen tension-PbtO_2_ gradient unrelated to varying cerebral perfusion pressure, suggesting diffusion limitation of oxygen delivery as a mechanism for cerebral hypoxia in those patients.

In the randomized trial by Okonkwo et al. in which adding PbtO_2_-guided care significantly reduced the time of exposure to cerebral hypoxia in patients with severe traumatic brain injury [33], the PbtO_2_ treatment protocol consisted of multiple physiological interventions to improve cerebral oxygen delivery, including increasing PaO_2_, PaCO_2_, and/or MAP, decreasing ICP, and performing red blood cell transfusion. However, our findings together with those of the aforementioned studies suggest that nonspecific interventions such as increasing FiO_2_ alone are not likely to sufficiently increase the PbtO_2_ level in cardiac arrest patients with cerebral hypoxia secondary to impaired cerebral microcirculation or diffusion limitation physiology [22, 53, 54]. We believe that therapeutic strategies targeting diffusion limitation physiology and/or impaired cerebral microcirculation may be needed to effectively treat or prevent post-ROSC cerebral hypoxia occurring in the absence of hypoxemia or hypotension. For example, intrathecal sodium nitroprusside administration may be an effective treatment for cerebral microcirculatory impairment after ROSC. We found that it reversed the cerebral microcirculatory impairment after ROSC in pilot experiments. Based on these results, we can postulate that correction of diffusion limitation physiology and/or impaired cerebral microcirculation improves the PbtO_2_ level itself, as well as the response of PbtO_2_ to supplemental oxygen. We are planning future investigations on the effect of therapeutic strategies that target diffusion limitation physiology and/or impaired cerebral microcirculation on cerebral microcirculation parameters and PbtO_2_, These studies could illuminate why the PaO_2_ had a limited capacity to explain PbtO_2_ variance in the present study, and also help identify effective treatments for treating post-ROSC cerebral hypoxia.

Many further questions regarding the PbtO_2_ after cardiac arrest should be addressed prior to clinical implementation of PbtO_2_-guided care. First, the impacts of varying MAP, PaO_2_, and ETCO_2_ on PbtO_2_ levels in other cerebral regions, or on global cerebral oxygenation, must be investigated. Since the cerebral measurements were restricted to a small area of the cerebral cortex in our study, our results cannot be extended to the entire brain. Experimental studies in rats have reported marked regional differences in CBF and cerebral oxygenation after cardiac arrest [19, 58]. Manole et al. investigated cerebral cortical and thalamic PbtO_2_ levels in immature rats subjected to 9 min and 12 min asphyxial cardiac arrest [19], and reported that cerebral cortical hypoxia occurred despite the presence of hyperoxia in the thalamus. Second, the impacts of varying MAP, PaO_2_, and ETCO_2_ on cerebral oxygenation should be studied in different cardiac arrest models, specifically regarding the duration and etiology of cardiac arrest. In the study by Manole et al. [19], the reduction in PbtO_2_ was greater, and the PbtO_2_ response to supplemental oxygen was less pronounced, after a longer duration of asphyxia cardiac arrest. In addition, several studies have reported significant differences in patterns of CBF reperfusion and cerebral injury between VF and asphyxial cardiac arrest [59, 60]. Third, although a number of studies have suggested the benefits of correcting cerebral hypoxia in traumatic brain injury [15–18, 33], studies assessing whether the correction of cerebral hypoxia is beneficial after cardiac arrest are limited [21, 22]. Therefore, further experiments assessing the relationship between PbtO_2_ and cerebral metabolic and neurophysiological markers after cardiac arrest are warranted. Additionally, further studies to determine the effects of correcting cerebral hypoxia on histopathological and functional neurological outcomes after cardiac arrest are crucial.

Our study has several important limitations. First, data were obtained from anesthetized animals free of an underlying disease. Despite similarities in neuroanatomy between pigs and humans, direct extrapolation of our findings to human cardiac arrest patients cannot be assumed. Second, we retrospectively analyzed data derived from a previous study. Our study could not determine causal relationships between the common target variables and PbtO_2_. Third, the data were obtained from a study conducted in a well-controlled experimental setting. Variation in factors related to cardiac arrest and resuscitation, including arrest etiology and duration of arrest, in the clinical setting may result in different results. Fourth, other factors influencing PbtO_2_, including ICP and oxygen consumption, could confound the relationship between the common target variables and PbtO_2_, but they were not included in our study.

## Conclusions

In the post-hoc analysis of data from an experimental study in which the animals did not experience hypoxemia or hypotension during the early post-resuscitation period, MAP and ETCO_2_ were not significantly related to PbtO_2_. The PaO_2_ level had a significant linear association with the PbtO_2_ level, but its ability to explain PbtO_2_ variance was small.

## Supporting information

S1 Movie Representative cerebral microcirculation video. It shows changes in cerebral cortical microcirculation during the first 15 min of the post-resuscitation period.
(MP4)

S1 Data Raw data. (XLSX)

S1 File ARRIVE guidelines checklist. (DOC)
